# Examining the reliability, correlation, and validity of commonly used assessment tools to measure balance

**DOI:** 10.1002/hsr2.98

**Published:** 2018-10-27

**Authors:** Nicole Dawson, Darcy Dzurino, Melissa Karleskint, Jennifer Tucker

**Affiliations:** ^1^ Division of Physical Therapy, School of Kinesiology and Physical Therapy University of Central Florida Orlando FL USA

**Keywords:** patient outcome assessment, postural balance, psychometrics

## Abstract

**Objectives:**

The Biodex SD Stability System has been shown to be a reliable assessment tool for postural stability. However, its ability to provide an accurate representation of balance has not been compared with functional performance measures such as the four‐square step test (FSST) and timed‐up‐and‐go test (TUG). The purpose of this study was to investigate reliability, internal consistency, and construct validity of FSST, TUG, and Biodex SD (limits of stability [LOS] and modified Clinical Test of Sensory Organization and Balance [m‐CTSIB]).

**Methods:**

An observational reliability and validity study was conducted. A convenience sample of 105 healthy adults, 77 females and 28 males, mean age 24.5 years old (± 4.66 SD) performed balance assessments including the FSST, TUG, Biodex SD LOS, and m‐CTSIB. For LOS, the overall percentage and test duration were recorded. For m‐CTSIB, the overall Sway Index was recorded. Condition 1 of the m‐CTSIB represented simple postural stability.

**Results:**

The Biodex SD LOS overall percentage, TUG, and FSST showed strong to excellent test‐retest reliability (ICC [3, 1] = .83 [mean 1: 58.14, mean 2: 60.54], .88 [mean 1: 6.98 seconds, mean 2: 6.91 seconds], .92 [mean 1: 6.29 seconds, mean 2: 6.14 seconds], respectively), while the Biodex SD m‐CTSIB overall percentage demonstrated strong test‐retest reliability (ICC [3, 1] = .75 [mean 1: 1.18, mean 2: 1.18]). The LOS test duration showed moderate test‐retest reliability (ICC [3, 1] = .58 [mean 1: 38.55 seconds, mean 2: 37.10 seconds]), while the m‐CTSIB condition 1 showed poor test‐retest reliability (ICC [3, 1] = .24 [mean 1: 0.63, mean 2: 0.66]). Weak construct validity was found between TUG, FSST, and Biodex SD measures of LOS and m‐CTSIB (*r* values = −0.15‐0.22).

**Conclusion:**

It is suggested that clinicians use more than one measure to assess different aspects of a patient's balance deficits to better guide treatment and intervention.

## 1 INTRODUCTION

Balance and postural control are essential to ensuring not only safe activities of daily living for individuals, but for the performance of safe locomotion in general. These two components of human performance serve as a foundation of stability prior to achieving more complex controlled mobility and skilled activities such as independent standing and walking.^1^ Postural control, or stability, represents an individual's capacity to maintain an upright position during both static and dynamic conditions, with or without the application of external perturbation or displacement of support surface.^1^, ^2^, ^3^, ^4^ Posture is an angular measure from vertical describing the gravitational vector of the body's orientation.^5^ Static balance is often defined as a person's ability to maintain control of their center of mass (COM) over a fixed base of support (BOS) while on a firm, flat surface. Even during static or quiet stance, researchers differ on the most important variables (eg, center of pressure, COM, difference between these variables^5^:), which can make it more complicated when attempting to select an assessment tool. Dynamic balance, on the other hand, refers to a person's ability to maintain postural control of their COM over a fixed BOS while either the surface is no longer firm or flat, or while the individual is reaching or performing other extremity movements while maintaining balance.^6^ Additionally, functional balance is a person's ability to maintain control of their COM over a moving BOS, or while performing a more complex controlled mobility or skilled activity.^7^

Pickerill and Harter^7^ identify three key problems with the current methods for balance assessment: nomenclature, criterion standards, and technology. The terms “balance” and “postural stability” are often considered interchangeable in rehabilitation sciences due to the lack of standardized nomenclature and operationalization. It is important that a clinician understands what aspect of balance is being assessed in order to make appropriate testing and treatment decisions. There is a lack of a single evaluative construct that defines good or normal dynamic balance.

Generally, in terms of balance and postural measures, there is a lack of reliability and validity data supporting the utilization of any one method as the best objective tool to capture a comprehensive balance component of a musculoskeletal and neuromuscular examination. This makes it extremely problematic for clinicians and researchers interested in postural stability assessment to not only accurately identify and adequately describe balance deficits at an initial examination or at baseline in a research study, but to be certain that the selected intervention or treatment provided adequate improvements in balance at the time of re‐assessment or follow‐up visit. Pickerill and Harter^7^ compared the Biodex Stability System dynamic limits of stability (LOS) test (Biodex Medical Systems, Shirley NY), which challenges patients to move and control their center of gravity within their BOS and is a good indicator of dynamic control within a normalized sway envelope,^8^ to neuroCOM smart balance master dynamic LOS test (Natus Medical Incorporated, Pleasanton CA). Authors found a low correlation between the two stability tests revealing they measure distinctly different constructs of postural stability. The concurrent and construct validity of either LOS test were not established by the aforementioned study, and these authors recommended further research aimed at repeating this study with a clinical population.^7^ Neither, however, was compared with commonly used clinical or functional dynamic balance measures such as timed‐up‐and‐go (TUG) and four‐square step test (FSST); therefore, it would be beneficial to examine the correlation between computerized posturography and clinical outcome measures, to identify the level of construct validity, if any, between common measures of dynamic balance. This would give clinicians and researchers more information regarding the properties of these various assessment tools to assist with determination of the most appropriate tools to select during an examination or screening.

Hinman (2000) describes the differences in test‐retest reliability of balance measures produced by the Biodex Balance System in a summary of four studies. In each study, subjects had to perform two 30‐second tests under varying conditions. Test‐retest reliability of the subjects' LOS and overall stability index (SI) were both computed. The interclass correlation coefficients (ICC) for the overall SI ranged from .44 to .89 for the static balance tests. The ICCs for the LOS tests, on the other hand, ranged from .64 to .89, demonstrating less variability than static measures. As these ranges are rather large, further research must be done to better establish the reliability of the LOS tests with the Biodex Stability System (Hinman, 2000).^9^

It should also be taken into consideration that these particular studies did not include comparison of the Biodex measures to more clinical measures such as the TUG or FSST. Identifying the construct validity of these Biodex measures and determining the association when compared with commonly used functional assessments will be pertinent to the clinical world. Clinicians need to have knowledge and understanding of the particular construct the tool used is actually measuring. This information is instrumental in the development of a plan of care and allows an accurate representation of a patient's baseline in order to guide treatment and intervention.

Balance is a “generic” term and serves as the foundation of stability prior to more controlled mobility, yet there is no “gold standard” for its measurement.^5^, ^7^, ^10^ Balance may be difficult to capture in a single assessment, as it is a complex construct with reliance on multiple afferent and efferent physiological systems including vision, somatosensory, and vestibular.^11^ As often times clinicians attempt to use a single assessment tool during the examination, it is, therefore, necessary to gather a more thorough understanding of which tools are best at providing the necessary information required for clinical decision making. Functional performance measures such as the FSST^12^ and the TUG^13^ have been found to have clinical utility when assessing various parameters, such as balance and postural control, in different planes of motion, while also being highly sensitive and specific in the assessment of fall risk. These tests are quick, efficient, and require minimal equipment or training. These measures have been easily implemented by physical therapists worldwide into routine musculoskeletal examinations.^13^ Although these measures have been used consistently in clinical practice, new methods of measuring these same variables, such as the Biodex Balance System and other devices using computer technology, have been introduced into rehabilitation settings.

**Figure 1:**
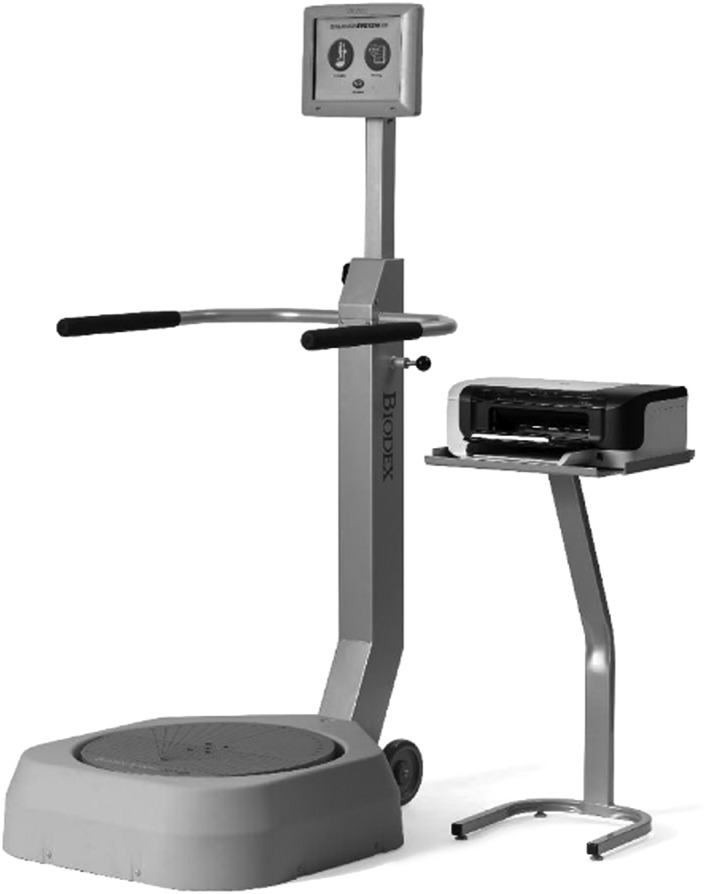
Biodex SD balance system

The Biodex Balance System SD (BBS: Biodex Medical Systems, Shirley NY) was introduced into research and clinical areas in the late 1990s (Figure 1). The BBS is a multi‐axial device used to quantitatively measure and record an individual's maintenance of posture during both static conditions and while under dynamic stresses. The BBS is equipped with a circular platform that can move in anterior, posterior, medial, and lateral directions. It is capable of producing clinical data measurement with application across many populations. Previous studies have shown its reliability as a tool for objective assessment of postural stability (1, 7; Hinman, 2000).^9^ However, its ability to provide an accurate representation of balance parameters is very limited in comparison to that of functional performance measures widely used now, such as the FSST and TUG.^1^ Research demonstrated that the Biodex SI was not correlated to the Y Balance Test in individuals with or without lower limb injuries.^2^ Commonly used rehab tools, such as the FSST and TUG, may be the most efficient and most accurate representation of a patient's balance; however, with the introduction of new technology, such as the Biodex Stability System, clinicians and researchers should examine if an update in standard of care is needed. The purpose of this study was to investigate reliability and construct validity of the Biodex Stability System SD (Modified Clinical Test of Sensory Interaction on Balance [m‐CTSIB] and LOS tests) as compared with more clinically available and common assessments (FSST and TUG). The FSST and TUG were chosen as they are measures of dynamic mobility and also good indicators of an individual's risk for falls.^12^, ^14^ This will allow clinicians and researchers to determine if the entire picture of a patient's balance can be captured with any of these assessment methods.

## 2 METHODS

### 2.1 Study design and subjects

This study was an observational, closed cohort design, and consisted of a convenience sample of 105 healthy adults, 77 females and 28 males, mean age 24.5 years old (± 4.66 SD), who met the specific inclusion criteria: at least 18 years old and generally healthy. Individuals were excluded from the study if they had any current musculoskeletal injury, visual impairment that affected their daily living, vestibular disorder, neurological disorder, cognitive disorder, and any further medical condition that would prohibit them from participating safely in the chosen balance measures. Subjects were recruited from the University of Central Florida, where the study was conducted between March 2016 and May 2016. The Institutional Review Board at the University of Central Florida approved this study (SBE‐16‐12078), and verbal informed consent was collected from all participants prior to the start of data collection.

### 2.2 Materials

Timed‐up‐and‐go (TUG)^13^:

The TUG is a widely used clinical performance‐based assessment tool used to measure an individual's lower extremity function, mobility, and fall risk. The TUG is able to correctly identify fallers and non‐fallers with 87% sensitivity and specificity, and has a suggested cutoff point of 13.5 seconds.^13^, ^14^

**Figure 2:**
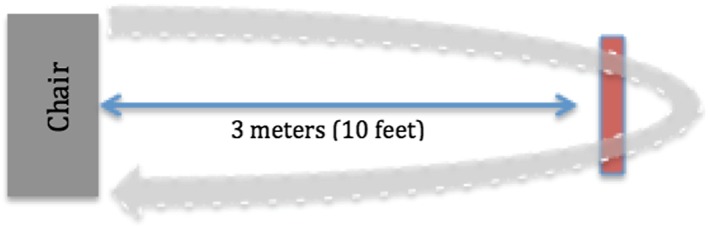
Timed‐up‐and‐go test

The participant was asked to start seated, with their back against a standard height chair, without armrests. At the start of the timer and the investigator's “Go!” command, the participant stood up from the chair and walked at a normal, comfortable pace for 10 feet (3 meters) to a line on the floor, where they turned around and returned to a seated position in the chair (see Figure 2). The investigator stopped the timer when the participant's buttocks touched the chair. Two trials were performed for each participant, and both times were recorded.

Four‐square step test (FSST)^12^:

The FSST is a test of dynamic balance that assesses a person's ability to step over obstacles in three directions of motion: forwards, backwards, and sideways. Populations tested by this assessment tool include geriatrics and those suffering from Parkinson's disease, stroke, transtibial amputation, and vestibular disorders.^15^, ^16^, ^17^, ^18^ A cut‐off score of 15 seconds serves as the threshold of older adults at risk for multiple falls, with a specificity of 88% and a sensitivity of 85%.

**Figure 3:**
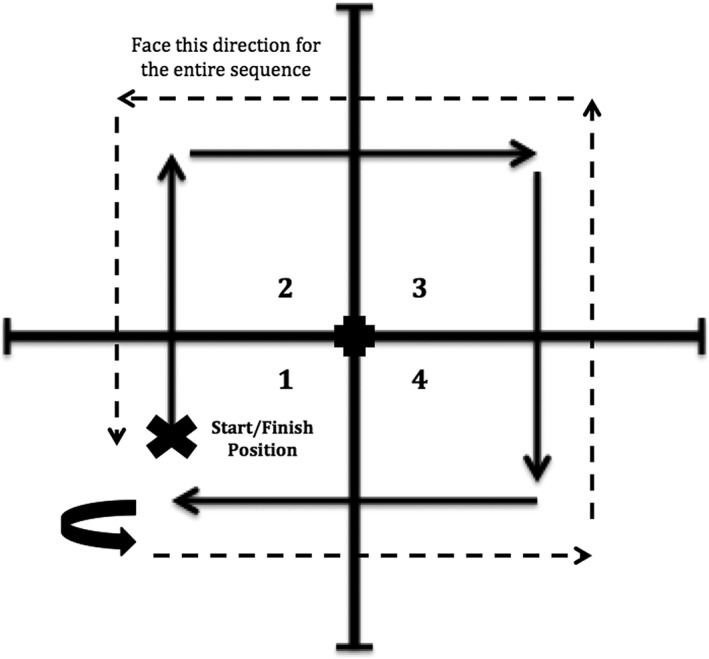
Four‐square step test

The test setup consisted of four canes of the same width in a cross formation (see Figure 3). The participant was instructed to step into each square, labeled 1 through 4, in a clockwise sequence: 2, 3, 4, 1, 4, 3, 2, and 1 (see Figure 2). The participant was asked to complete the sequence as fast as possible without hitting the equipment. Each subject was allotted one practice run if necessary, and then two trials were performed and recorded for each participant. A lower time recorded in seconds reflected better performance on this measure.

Biodex Balance System SD:

The Biodex Balance System SD features five test protocols and six training modes, allowing for both testing and training in either static or dynamic formats. It is intended to be a system that can provide accurate Fall Risk Screening and Conditioning for older adults, can be used as a balance assessment tool for concussion management, and can serve as weight‐bearing assessment and training for lower extremity injuries.^8^ Neuromuscular control can be assessed by the device's ability to quantify the patient's ability to maintain static and dynamic postural stability on a stable or unstable surface. The Biodex System consists of a platform that features both static and dynamic balance capabilities, adjustable support handles, a 12.1″ high‐resolution color touch‐screen LCD display, and a color printer with stand for printing results of testing assessments. Two different testing protocols were used in this study: The LOS test and the modified‐Clinical Test of Sensory Organization and Balance (m‐CTSIB).

1. Limits of stability (LOS):

The LOS test challenges the participants to move and control their COM to remain within their BOS. It serves as an indicator of dynamic control within a normalized sway envelope. An individual's LOS for standing balance is the maximum angle that body can achieve from vertical without losing balance. Once an individual exceeds their individual LOS, a fall, stumble, or step may ensure. The LOS in normal adults is defined as eight degrees anterior, four degrees posterior, and 16 degrees in the lateral direction.^19^ In this study, the default setting for the LOS test, which is 75% LOS, was used. This reflects a moderate skill level.

**Figure 4:**
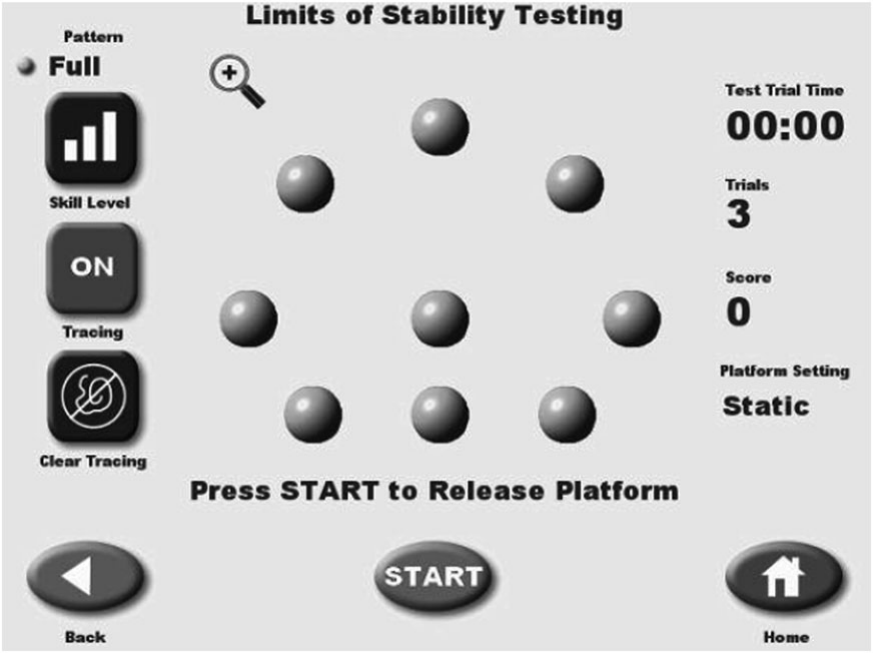
Limits of stability test (Biodex SD balance)

Once the participant was in correct foot position as defaulted on the screen, the investigator selected the “Limits of Stability Test,” as prompted. The test was explained to the participant as they were instructed to shift their weight to move a cursor toward each red, blinking target as displayed on the screen as quickly and with as much accuracy as possible. The nine targets were positioned in a circular fashion (see Figure 4), which required the individual to shift their weight toward a target in the periphery and then return to a central location prior to shifting their weight to the next target in the various patterns defaulted on the screen. The individual's feet were to remain planted and they were instructed to not use the handrails, except in case of safety concerns. Two trials were completed for each participant, and the results were printed and recorded. Test duration and overall percentage were recorded for each trial for each participant. Test duration reflected the total amount of time it took the individual to complete the blinking dot pattern. Overall percentage was defined as the amount of direction control accuracy the individual had when performing the test. If a participant were to score a 100%, this would suggest that the individual moved in a precise and straight line to each target; higher percentage thus reflected better performance. The overall percentage itself represents the amount of deviation from a straight pathway to the targets.

- Biodex Balance System SD modified Clinical Test of Sensory Organization and Balance (m‐CTSIB):

This test has been well documented in the literature as an effective test for identifying individuals with mild to severe balance deficits, as it also isolates which system is impaired.^20^, ^21^ The test protocol is meant to provide a generalized assessment of an individual's ability to both integrate various senses with respect to balance and to compensate when one of more of these senses has deficits.

This test included four conditions: (1) Eyes Open, Firm Surface, (2) Eyes Closed, Firm Surface, (3) Eyes Open, Foam Surface, and (4) Eyes Closed, Foam Surface. Each condition lasted 30 seconds, and a 10‐second rest break was allotted in between, if requested by the participant. During conditions 3 and 4, a Foam Mat (provided with the Biodex system) was placed onto the platform that contained the same markings as the firm surface, allowing the participant to reposition their feet into their previously instructed placement. For each of the four conditions, each participant was instructed to stand as still as they possibly could for the entire 30 seconds. Each condition was performed twice. The Overall Sway Index (SI) was recorded for each condition of each trial, which summed the average of each condition. The SI represented the standard deviation of the SI; therefore, the higher the SI, the more unsteady or unbalanced a person was during the test. Higher scores on the stability indices demonstrate a greater amount of postural sway or variability during that particular condition. This can also be interpreted as decreased postural stability. The SI for Condition 1 was also recorded separately as a measure of standard postural stability with the condition being Eyes Open and on a Firm Surface. After each of the two trials, the results were printed and recorded.

### 2.3 Procedure

Participants attended a single session for data collection, which consisted of a short demographic questionnaire (eg, age, highest education received) and a battery of balance assessments, including the FSST, TUG, Biodex SD LOS, and Biodex SD m‐CTSIB.

Each of the participants performed the four balance assessments. First, each subject performed two trials of the TUG. Next, each study participant was allotted one practice trial of the FSST prior to the performance of two timed trials. Next, the participant performed two separate balance assessments on the Biodex Balance System SD. Each participant's feet were positioned onto the platform using the default values based on their individual height. The first test to be performed was the LOS. The default setting for this test is 75% LOS (moderate still level). Limits of stability hold times were defaulted to 0.25 seconds, and each rest countdown in between trials lasted 3 seconds. Two trials were performed. Finally, each participant performed the m‐CTSIB. The participant assumed the same foot position as they did in the LOS test. Each of the four conditions lasted for 30 seconds, and two trials of all four conditions were performed.

### 2.4 Statistical analysis

Data analysis was performed with SPSS Statistical Software (version 22, IBM Statistics), and preparatory analyses were conducted. All outcome variables were found to have a normal distribution via Shapiro‐Wilk testing with the exception of the Biodex SD m‐CTSIB, which demonstrated moderate positive skewness. Descriptive statistics were determined for demographics, while the Biodex Balance System SI and LOS scores were calculated by the computer interface included with the system.

Intraclass correlation coefficients (ICC [3, 1]) were used to determine test‐retest reliability for each of the six variables (FSST, TUG, Biodex SD LOS test duration, Biodex SD overall percentage, Biodex SD m‐CTSIB condition 1, and Biodex SD m‐CTSIB SI). Since ICC [3, 1] is considered a model of analysis of variance, it is robust against the positive skewness of the Biodex SD m‐CTSIB variable.^22^, ^23^, ^24^ Interpretation of ICC values was based on guidelines offered by Portney and Watkins,^25^ with values above 0.75 being classified as good reliability, and those below 0.75 were classified as moderate to poor reliability. In accordance with Carter, Lubinsky, and Domholdt,^26^ Pearson's correlation, *r*, was calculated to determine construct validity of each measure to identify whether they assessed similar or unique components of balance. A high, positive Pearson value would indicate that high scores on one measure are correlated with high scores on another measure, and the same would be true for low scores between measures.^26^

## 3 RESULTS

Table 1 summarizes the reliability results. Biodex SD LOS overall percentage, TUG, and FSST showed strong to excellent test‐retest reliability (ICC [3, 1] = .83, .88, .92, respectively), while the Biodex SD m­CTSIB SI demonstrated strong test‐retest reliability (ICC [3, 1] = .75), indicating that these assessments can be repeated and still have reproducible results. The LOS test duration showed moderate test‐retest reliability (ICC [3, 1] = .58), and the m­CTSIB condition 1 showed poor test‐retest reliability (ICC [3, 1] = .24), indicating that these measures do not demonstrate good repeatability when it comes to using these assessments to score the same subject.

**Table 1:** Test‐retest reliability via interclass correlation coefficient

Balance Assessment	Mean 1 (SD)	Mean 2 (SD)	ICC (3,1)	Descriptor^25^
Timed up and go (seconds)	6.98 (0.97)	6.91 (0.93)	0.88	Strong
Four square step test (seconds)	6.29 (1.13)	6.14 (1.19)	0.92	Excellent
Biodex SD LOS—Overall percentage	58.14 (14.23)	60.54 (13.68)	0.83	Strong
Biodex SD LOS—Test duration (seconds)	38.55 (6.60)	37.10 (5.73)	0.58	Moderate
Biodex SD m‐CTSIB—Stability index	1.18 (0.26)	1.18 (0.26)	0.75	Strong
Biodex SD m‐CTSIB—Condition 1	0.63 (0.31)	0.66 (0.24)	0.24	Poor

Abbreviations: ICC, intraclass correlation coefficient; LOS, limits of stability; m‐CTSIB, Modified Clinical Test of Sensory Interaction in Balance; m‐CTSIB condition 1, Modified Clinical Test of Sensory Organization and Balance condition 1 (flat surface, eyes open).

Regarding the validity of the various measures, intercorrelations (using Pearson's *r*) between measures (Table 2) ranged from −.15 to .22, indicating poor construct validity among all measures, suggesting that these assessments are measuring completely different aspects of balance and are not able to be used interchangeably to determine a patient's balance assessment.

**Table 2:** Intercorrelations (Pearson's r) between Biodex SD and clinical tests

	TUG	FSST	Biodex LOS	Biodex m‐CTSIB Stability Index	Biodex m‐CTSIB Condition 1
Timed up and go	1				
Four square step test	.14	1			
Biodex SD LOS—Overall percentage	−.07	−.15	1		
Biodex SD m‐CTSIB—Stability index	.22*	.14	−.09	1	
Biodex SD m‐CTSIB—Condition 1	.09	.21*	−.01	.41**	1

Abbreviations: LOS, limits of stability; m‐CTSIB, Modified Clinical Test of Sensory Organization and Balance; m‐CTSIB condition 1, Modified Clinical Test of Sensory Organization and Balance condition 1 (flat surface, eyes open). Pearson's correlation:

* *P* < .05.

** *P* < .001.

## 4 DISCUSSION

Clinicians have used the terms “balance,” “postural stability,” and “sway” relatively interchangeably for the past few decades. As new assessment methods are introduced into clinical practice, there is a clinician demand to establish a single evaluation construct that defines balance. Many current methods for the objective assessment of balance were developed for special populations such as neurologic patients or older adults.^27^ Operationalizing the concept of dynamic postural stability remains elusive and sometimes depends on the specific field of study. Dynamic postural stability represents the ability of an individual to maintain balance while shifting their COM over a mobile BOS.^5^, ^11^ There are many definitions for balance and postural control. Theoretically, objective tools to measure balance should have variables that correlate highly, or at least moderately, with one another, to demonstrate construct validity of an assessment of an individual patient's balance. However, the commonly used assessment measures that were examined in the current study revealed poor construct validity, indicating that each tool assessed unique components of postural stability and balance in this sample of participants. Additionally, reliability of these balance tests should demonstrate a level of repeatability that promotes confidence in the utilization of these tools for the clinician or researcher wanting to perform a complete dynamic postural stability assessment. The current study found that five of the six measures demonstrated moderate, strong, or excellent reliability, the exception being the Biodex SD m‐CTSIB—Condition 1. The results, however, left uncertainty regarding the particular construct being measured.

These findings contribute significantly to the current body of literature, as results demonstrate strong to excellent test‐retest reliability for the TUG, FSST, Biodex SD LOS—Overall Percentage, and Biodex SD m‐CTSIB—SI. These results support previous literature identifying excellent test‐retest reliability for FSST and TUG^12^, ^13^ while strengthening reliability data of Biodex SD LOS and m‐CTSIB tests. More interestingly, the study revealed poor construct validity between measures, indicating that the selected methods of balance assessment examine unrelated constructs. This supports previous findings by Almeida et al^2^ that identified no significant correlation between the Y Balance Test and the Biodex SI. These findings support the growing body of evidence that the constructs of balance and postural stability are complicated and involve a series of unique motor systems that require a focused examination by a skilled clinician. Sousa et al^10^ discuss the role of both efferent and afferent information required to maintain upright standing in various conditions; therefore, the study supports the need for a holistic examination.

Additionally, the current study provides additional normative data for a younger adult population on these measures. The TUG performance in the current study was slightly faster than previously established norms (Kear, Guck & McGaha, 2016), but it is possible that the sample in the current study had higher performance due to being healthy, active college graduate students rather than typical adults. No normative data are available for the FSST, the Biodex Balance SD LOS, or Biodex Balance SD m‐CTSIB in this age population; therefore, the current study provides that for future comparisons.

Findings from this study have clinical implications warranting further discussion in efforts to assist clinicians and researchers in selecting the most appropriate tool for the situation. Clinicians and researchers should be deliberate when choosing a balance assessment tool. For example, if a clinician wants to assess a more dynamic or functional component of balance, a TUG would be a quick, efficient, and reliable method to determine this aspect of an individual patient's balance during ambulation. On the other hand, the FSST has the ability to not only assess dynamic balance, but to also examine the coordination, directional change, and cognitive components of balance not necessarily looked at in the TUG. The Biodex Stability System is an expensive piece of equipment that does offer insight into different components of balance that are not necessarily able to be observed by a clinician with the naked eye, such as magnitude of a SI or degree of postural sway. For example, this specialized equipment could be recommended if a clinician wants to determine an objective SI between varying conditions to determine which system is affecting their balance ability (eg, proprioception, vision, vestibular), or to produce a computerized report to document improvements in a patient following rehabilitative interventions.

### 4.1 Limitations and future research

While these findings contribute significantly to the understanding of postural stability and balance, it should be noted that the study included a fairly homogenous sample of healthy adults; therefore, generalization of these results to clinical populations or samples of varying ages may not be appropriate. Additionally, it should be noted that the testing ordered was maintained for all participants; therefore, there is a potential for practice effects between trials of the assessments.

Future areas of research include the replication of this study with subjects in varying patient populations, for instance in a geriatric or neurologic population.^28^ For example, Brandmeir and colleagues (2015) studied falls and multiple outcome measures including the TUG, Berg Balance Scale, and Biodex SI in patients with Parkinson's disease. It is also important to recognize the different uses for these balance measures. Additionally, a more comprehensive battery of balance and mobility measures should be examined to understand the potential utility and relationship of each measure, to allow clinicians to have the most information possible to inform clinical decision‐making. Further research must be conducted to understand the exact use of each of these available balance assessments. Furthermore, similar studies broader assessments such as the Berg Balance Scale^29^ or the Fullerton Advanced Balance Scale^30^ may be interesting to provide clinicians with additional information for choosing the best assessment tools during an examination.

## 5 CONCLUSION

Balance is a complex construct, and it is recommended that clinicians understand this, as we encourage the utilization of multiple balance assessment tools to capture the entire picture of an individual's balance. Based on results of this study, it is suggested that clinicians use more than one balance test to assess different aspects of balance based on patient deficits to better guide treatment and intervention. It is important to take into account that while all of these outcome measures do look at components of balance, none of them can serve as a complete, single evaluative construct of balance itself.

## CONFLICTS OF INTEREST

The authors have declared that there is no conflict of interest.

## AUTHOR CONTRIBUTIONS

Conceptualization: Nicole Dawson, Jennifer Tucker

Data Curation: Nicole Dawson, Melissa Karleskint, Darcy Dzurino

Formal Analysis: Nicole Dawson

Investigation: Melissa Karleskint, Darcy Dzurino, Nicole Dawson, Jennifer Tucker

Project Administration: Nicole Dawson, Jennifer Tucker

Supervision: Nicole Dawson, Jennifer Tucker

Writing—Original Draft Preparation: Darcy Dzurino, Melissa Karleskint, Nicole Dawson, Jennifer Tucker

Writing—Review and Editing: Nicole Dawson, Jennifer Tucker
